# Distribution and inter-regional relationship of amyloid-beta plaque deposition in a 5xFAD mouse model of Alzheimer’s disease

**DOI:** 10.3389/fnagi.2022.964336

**Published:** 2022-07-28

**Authors:** Ka Chun Tsui, Jaydeep Roy, Sze Chun Chau, Kah Hui Wong, Lei Shi, Chi Him Poon, Yingyi Wang, Tatyana Strekalova, Luca Aquili, Raymond Chuen-Chung Chang, Man-Lung Fung, You-qiang Song, Lee Wei Lim

**Affiliations:** ^1^School of Biomedical Sciences, Li Ka Shing Faculty of Medicine, The University of Hong Kong, Pokfulam, Hong Kong SAR, China; ^2^Department of Anatomy, Faculty of Medicine, Universiti Malaya, Kuala Lumpur, Malaysia; ^3^Department of Neuroscience, Maastricht University, Maastricht, Netherlands; ^4^Department of Normal Physiology and Laboratory of Psychiatric Neurobiology, Sechenov First Moscow State Medical University, Moscow, Russia; ^5^Discipline of Psychology, College of Science, Health, Engineering, and Education, Murdoch University, Perth, WA, Australia; ^6^The State Key Laboratory of Brain and Cognitive Sciences, The University of Hong Kong, Pokfulam, Hong Kong SAR, China

**Keywords:** Alzheimer’s disease, amyloid-beta (AB), morphology, neuroanatomy, 5xFAD, dementia

## Abstract

Alzheimer’s disease (AD) is the most common form of dementia. Although previous studies have selectively investigated the localization of amyloid-beta (Aβ) deposition in certain brain regions, a comprehensive characterization of the rostro-caudal distribution of Aβ plaques in the brain and their inter-regional correlation remain unexplored. Our results demonstrated remarkable working and spatial memory deficits in 9-month-old 5xFAD mice compared to wildtype mice. High Aβ plaque load was detected in the somatosensory cortex, piriform cortex, thalamus, and dorsal/ventral hippocampus; moderate levels of Aβ plaques were observed in the motor cortex, orbital cortex, visual cortex, and retrosplenial dysgranular cortex; and low levels of Aβ plaques were located in the amygdala, and the cerebellum; but no Aβ plaques were found in the hypothalamus, raphe nuclei, vestibular nucleus, and cuneate nucleus. Interestingly, the deposition of Aβ plaques was positively associated with brain inter-regions including the prefrontal cortex, somatosensory cortex, medial amygdala, thalamus, and the hippocampus. In conclusion, this study provides a comprehensive morphological profile of Aβ deposition in the brain and its inter-regional correlation. This suggests an association between Aβ plaque deposition and specific brain regions in AD pathogenesis.

## Introduction

Alzheimer’s disease (AD) is a neurodegenerative disorder characterized by the deposition of amyloid beta (Aβ) plaques and the aggregation of neurofibrillary tangles (NFTs) caused by the hyperphosphorylated tau protein, and involves mutations in amyloid precursor protein (APP), presenilin 1 (PSEN1), presenilin 2 (PSEN2), and apolipoprotein E (APOE) (Bekris et al., 2010; Iqbal et al., 2010; Poon et al., 2020). Patients with AD generally experience memory deficits, cognitive impairment, psychological changes, and sleep disorders. It is the most common form of dementia and affects over 55 million people worldwide, with healthcare costs estimated at $355 billion US dollars in 2021 (Bature et al., 2017; WHO, 2019). Despite extensive research, there is still no effective treatment on the market mainly due to our limited understanding of AD pathogenesis. The prevailing hypothesis is that the deposition of Aβ plaques causes a cascade of neurotoxic events that result in observable synaptic and neuronal loss with neurotransmission dysfunction, eventually leading to AD symptoms (Kametani and Hasegawa, 2018; Soria Lopez et al., 2019; Wong et al., 2020).

Several transgenic models have been developed to overexpress mutant forms of the amyloid precursor protein to mimic AD pathologies such as Aβ deposition. The transgenic 5xFAD mouse model was developed in 2006 to study late-onset AD. This mouse model consists of five familial AD mutations: Swedish APP mutation (K670N/M671L), Florida APP mutation (I716V), and London APP mutation (V717I), PSEN1 M146L mutation, and the PSEN1 L286V mutation (Poon et al., 2020; Forner et al., 2021). These mutations result in the overproduction of Aβ peptides, leading to observable Aβ deposition in the brain as early as 1.5 months (Poon et al., 2020; Forner et al., 2021) and memory deficits by 4–6 months (Eimer and Vassar, 2013; Poon et al., 2020). Although previous studies have selectively reported the deposition of Aβ plaques in specific brain regions in animal models of AD (Oakley et al., 2006; Rodrigue et al., 2009; Vlassenko et al., 2012; Oh et al., 2018; Kim et al., 2020), a comprehensive investigation which gather all the distribution information of Aβ plaques across the brain has not been conducted. Our study aims to provide a comprehensive distribution profile of the rostro-caudal deposition of Aβ and its inter-regional correlation in adult 5xFAD brain.

## Materials and methods

### Subjects

Nine-month-old female 5xFAD mice (*n* = 10) and C57bL/6J wildtype (WT) mice (*n* = 9) were socially housed in standard cages (4–5 mice per cage) with access of food and water *ad libitum*. The female 5xFAD mouse model was selected based on the sex-biased epidemiological profile and neuropathological development in women compared to men (Bundy et al., 2019; Sil et al., 2022). The mice were kept under a 12-h light/dark cycle under controlled temperature (22 ± 1°C) and humidity (60–70%). The procedures for behavioral testing and euthanasia were approved by the Committee on the Use of Live Animals in Teaching and Research, the University of Hong Kong (No. 4807-18).

### Forced alternation Y-maze

The experimental procedure was conducted as previously described to measure working memory function (Melnikova et al., 2006; Yu et al., 2022). In brief, animals were placed in a symmetrical Y-maze (each arm: 35 cm × 35 cm × 35 cm) in dim light condition (30 ± 5 lux). The test consisted of an acquisition phase and a retrieval phase (5 min each). During the acquisition phase, the animal was placed in the start arm and allowed to explore only two arms of the Y maze, with the third arm blocked. The retrieval phase was performed 30 min after the acquisition phase. During the retrieval phase, the animal was placed in the start arm and allowed to explore all three arms. The time spent in the novel arm and the total distance traveled in the retrieval phase was recorded and analyzed using ANY-maze software (Stoelting Co., Wood Dale, IL, United States).

### Open field test

The experiment was conducted as previously described to evaluate locomotor function (Lim et al., 2016; Tan et al., 2020b). The test was conducted in an enclosed square arena (40 cm × 40 cm × 40 cm) in dim light condition (30 ± 5 lux). Animals were placed in the middle of the area and allowed to explore freely for 5 min. The total distance traveled was recorded by video and subsequently measured using ANY-maze software (Stoelting Co., Wood Dale, IL, United States).

### Morris water maze

This test was conducted to measure the hippocampal-dependent learning and memory function as previously described (Liu et al., 2015; Tan et al., 2020a; Yu et al., 2022). The apparatus consisted of a black circular pool (150 cm × 60 cm) filled with water at 25 ± 1°C. The water in the pool was colored opaque using non-toxic skim milk powder. A circular platform (11 cm diameter) was placed 1 cm below the water surface and 15 cm away from the pool wall. The experiment was conducted in dim light condition (30 ± 5 lux). The spatial acquisition consisted of training phase 1 (day 1–4), and training phase 2 (reversal phase, day 6–7). The spatial acquisition training phase 1 consisted of four trials per day (1 min duration with 90 s interval) for four consecutive days (day 1–4). In the training phase, mice were trained to locate the submerged platform in the black circular pool with randomized starting positions that were equidistant from the submerged platform. If the mice were unable to locate the platform, they were gently guided onto the platform. On day 5, a probe test was carried out 24 h after day 4 to assess long-term memory function. After the probe test, the reversal phase consisted of four consecutive trials (1 min each with 90 s interval) carried out on day 6–7. In the reversal phase, the submerged platform was placed in the opposite quadrant of the water maze and each animal was trained with randomized starting positions. On day 7, another probe test was performed to measure the short-term memory function with the platform removed 90 min after the last trial. All trials were video recorded and the frequency to enter each imaginary quadrant and the time spent in each quadrant were evaluated by ANY-maze software (Stoelting Co., Wood Dale, IL, United States).

### Histological study

All animals were anesthetized with overdose of sodium pentobarbital and perfused transcardially with Tyrode solution (0.8% sodium chloride, 0.02% potassium chloride, 0.0005% magnesium chloride hexahydrate, 0.1% sodium bicarbonate, 0.004% sodium phosphate monobasic, and 0.1% glucose) and 4% paraformaldehyde. Brain samples were harvested and post-fixed for 24 h in 4% paraformaldehyde, followed by incubation in 15 and 30% sucrose solutions until brains sank to the bottom. The brains were snap-frozen in liquid nitrogen before storing at −80*^o^*C.

The immunohistochemistry procedures were performed as previously described with minor modifications (Lim et al., 2015b; Liu et al., 2015). Brains were sliced into 20-μm coronal sections (Bregma: from 4.3 to −8.0 mm) using a CryoStar NX50 Cryostat (Thermo Fisher Scientific, Waltham, MA, United States). All sections were washed in PBS and incubated with 3% H_2_O_2_ in 0.01 M phosphate buffered saline and 0.5% Triton X-100 (PBS-T) for 15 min, and then incubated in 1% bovine serum albumin for 15 min. A primary mouse anti-human 4G8 antibody (1:500, Biolegend, CA, United States) was added and sections were incubated at 4*^o^*C for 24 h. After rinsing, secondary biotinylated goat anti-mouse antibody (1:500, Vector Laboratories, CA, United States) was added and sections were incubated at room temperature for 90 min, followed by avidin and biotinylated horse radish peroxidase (1:1,000, Vectastain, Vector Laboratories, CA, United States) and further incubated for 2 h. Finally, sections were incubated in 1 mg/mL 3,3′-diaminobenzidene tetrahydrochloride (DAB Substrate Kit; Vector Laboratories, CA, United States) in Tris–HCl with 0.005% H_2_O_2_ and 8% nickel ammonium sulphate. After dehydration, sections were mounted and cover-slipped with Permount (Thermo Fisher Scientific, Waltham, MA, United States). Color images were acquired under a Zeiss AxioPhot upright microscope (ZEISS, Germany) at 5× magnification.

### Evaluation of Aβ plaque deposition in different brain regions

The level of Aβ plaque deposition was qualitatively graded by two independent researchers according to the intensity level (0 = no deposition; 1 = mild; 2 = moderate; 3 = high). The methodology of intensity evaluation was conducted as previously described (Chong et al., 2019). The evaluation was conducted with researchers blind to the experimental design. The inter-rater reliability score was calculated as 93.2%. Representative photomicrographs of each intensity category of Aβ plaque deposition are presented in Figure 2C. Mild intensity was described as sparse Aβ deposits, moderate intensity was described as scattered clusters of Aβ deposits, and high intensity was described as dense clusters of Aβ deposits. The Aβ burden (*n* = 4–6 mice, around 12–48 sections) in each selected regions of interest (ROIs) were quantified using ImageJ (NIH, Bethesda, MD, United States) using the threshold analysis method. In this method, we first use the free hand selection tool to outline the entire region, then the image was divided into two classes of pixels, the foreground Aβ plaque and the background (Schneider et al., 2012).

### Correlation matrix and 3D model

Correlational analysis was used to measure the correlation as previously described (Wheeler et al., 2014). Briefly, the Aβ plaque burden was the dependent variable, which was averaged for each ROI. We then calculated the pairwise Pearson correlation coefficients between all ROIs to construct a symmetric correlation matrix. High correlation between pairs were noted when Aβ plaque burden in one region was strongly related to another region. Each Pearson correlation coefficient was displayed on the color-coded correlation matrix using GraphPad Prism 9.0 (GraphPad Software, San Diego, CA, United States). A 3D model was constructed by only considering the strongest inter-regional correlations. To determine the correlation threshold, we only retained correlations with a *p*-value less than 0.05, corresponding to a network correlation coefficient higher than ± 0.9. The resulting 3D model was visualized in R using the cocoframer package (Lein et al., 2007; Oh et al., 2014).

### Statistical analysis

All data analyses were performed in IBM SPSS Statistics 27. The results were reported as the mean ± S.D, unless otherwise indicated. Kolmogorov-Smirnov test was performed to examine data normality. The Y-maze and OFT results were analyzed by independent samples *t*-test. The Morris Water Maze (MWM) training latency was analyzed by factorial mixed design ANOVA for day 1–7. Inter-reliability analysis was performed to qualitatively assess the reliability of the intensity grading by the two researchers. Finally, Pearson correlation coefficients were calculated to examine the inter-regional correlation of the Aβ plaque deposition.

## Results

### Spatial and learning memory deficits in 5xFAD mice

**FIGURE 1:**
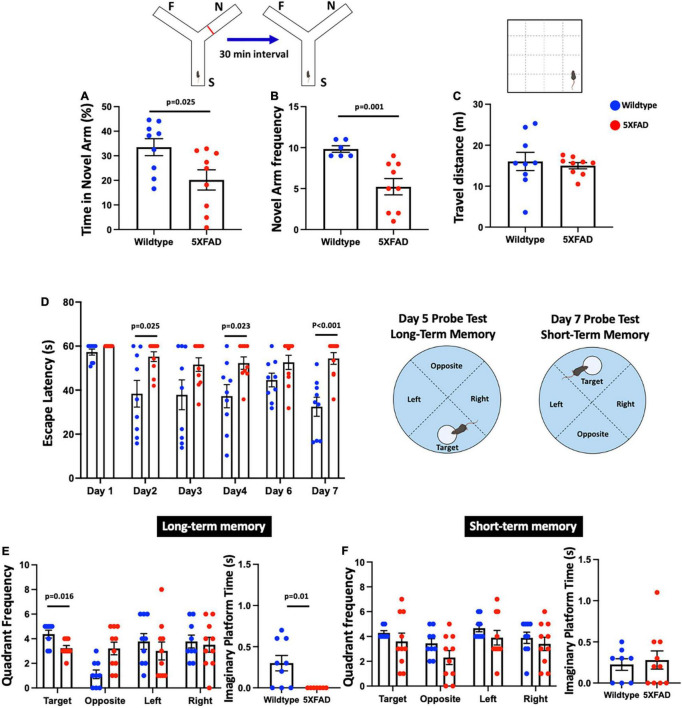
A symmetrical Y-maze experiment was conducted with Start Arm (S), Familiar Arm (F), Novel Arm (N). 5xFAD mice showed **(A)** significantly decreased proportion of time spent (%) in the novel arm, **(B)** significantly decreased frequency of visits to the novel arm, **(C)** no significant difference in the distance traveled compared to wildtype mice, **(D)** significantly increased escape latency (s) on days 2, 4, and 7, but no significant differences on days 1, 3, and 6, **(E)** significant decreased frequency to enter the target quadrant and time spent on the platform in the long-term memory probe test, and **(F)** no significant difference in the short-term memory probe test.

In the forced alternation Y-maze test, we found a significant reduction in the time spent in the novel arm [*t*_(16)_ = 2.477, *p* = 0.025] and the frequency to enter the novel arm [*t*_(16)_ = 4.291, *p* = 0.001] in 5xFAD mice compared to the wildtype control (Figures 1A,B). In the OFT, we found no difference in the distance moved [*t*_(16)_ = 0.445, *p* = 0.662] between 5xFAD mice and wildtype mice (Figure 1C), indicating locomotor function was not impaired. In the MWM, repeated-measures ANOVA revealed significant main effects for day [*F*_(5_,_75)_ = 6.066, *p* < 0.001], group [*F*_(1_,_15)_ = 12.076, *p* = 0.003], and their interaction [*F*_(5_,_15)_ = 3.095, *p* = 0.014] (Figure 1D). Interestingly, there was a significant reduction in learning memory with increased escape latency in 5xFAD mice on days 2, 4, and 7 [all *t*_(16–17)_ = < −2.452, *p* < 0.025], but no significant differences were observed on days 1, 3, and 6 [all *t*_(16–17)_ = < −1.787, *p* > 0.082] compared to wildtype mice. In the MWM probe test for long-term memory function, there was a significant decrease in the frequency to enter the target quadrant in 5xFAD mice [*t*_(15)_ = 2.722, *p* = 0.016] compared to wildtype mice, but no significant differences were found for the other quadrants [all *t*_(17)_ = 1.085, *p* > 0.293] (Figure 1E). The Mann-Whitney *U* test revealed a significant reduction in the time spent on the platform in 5xFAD mice (*Z* = −2.599, *p* = 0.010) compared to wildtype mice. We found no significant differences in the latency to escape in MWM probe test for short-term memory function [all *t*_(15–17)_ = < −1.787, *p* > 0.115] (Figure 1F).

### Aβ plaque deposition in different brain regions in 5xFAD mice

**FIGURE 2:**
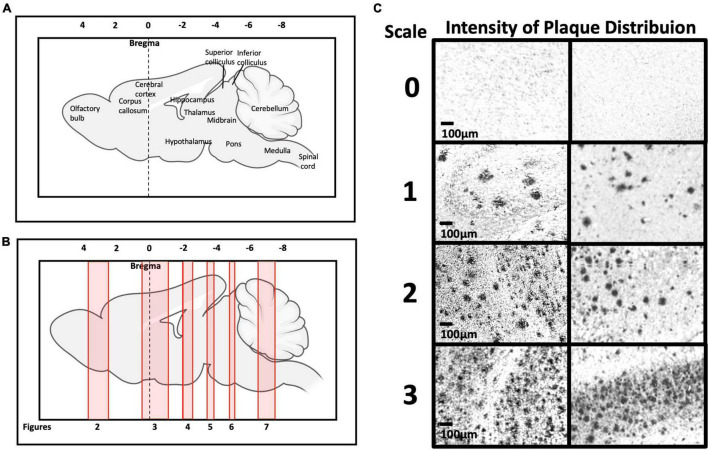
**(A,B)** Representation of brain rostro-caudal levels of Aβ deposition within the respective coronal brain sections depicted in Figures 2–8. **(C)** Categorization of Aβ deposition according to the intensity level (0 = no deposition; 1 = mild; 2 = moderate; 3 = high).

**FIGURE 3:**
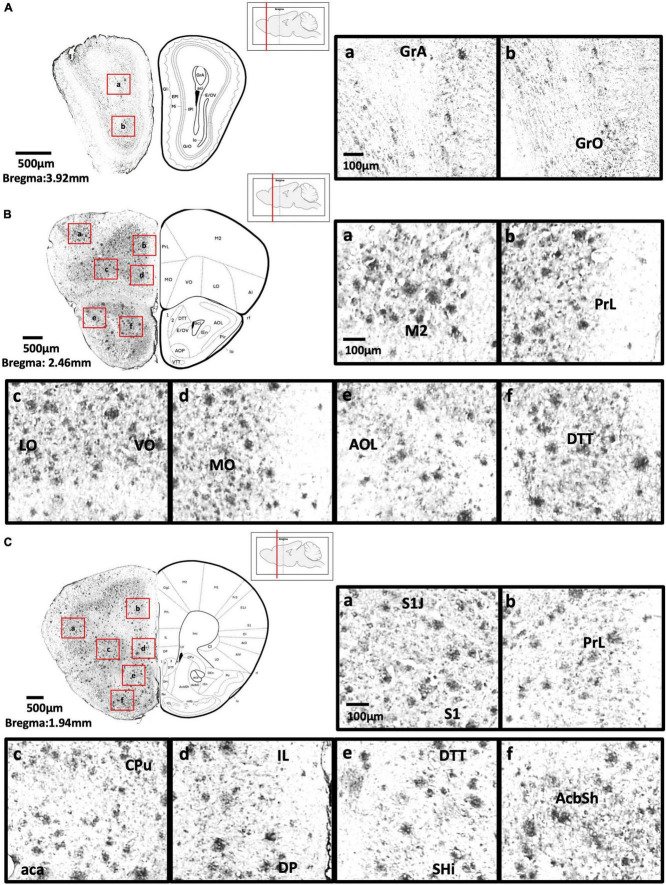
**(A)** Coronal section of olfactory-related areas showing Aβ deposition in the granule cell layer of the accessory olfactory bulb (a) and granule cell layer of the olfactory bulb (b). **(B)** Coronal section of the frontal cortex and olfactory-related areas showing Aβ deposition in the secondary motor cortex (a), prelimbic cortex (b), lateral orbital cortex, ventral orbital cortex (c), medial orbital cortex (d), lateral part of anterior olfactory area (e), and dorsal tenia tecta (f). **(C)** Coronal section through the cerebral cortex showing Aβ deposition in the primary somatosensory cortex, jaw region of the somatosensory cortex (a), prelimbic cortex (b), caudate putamen, anterior part of anterior commissure (c), infralimbic cortex, dorsal peduncular cortex (d), dorsal tenia tecta, septohippocampal nucleus (e), and nucleus accumbens shell (f).

**FIGURE 4:**
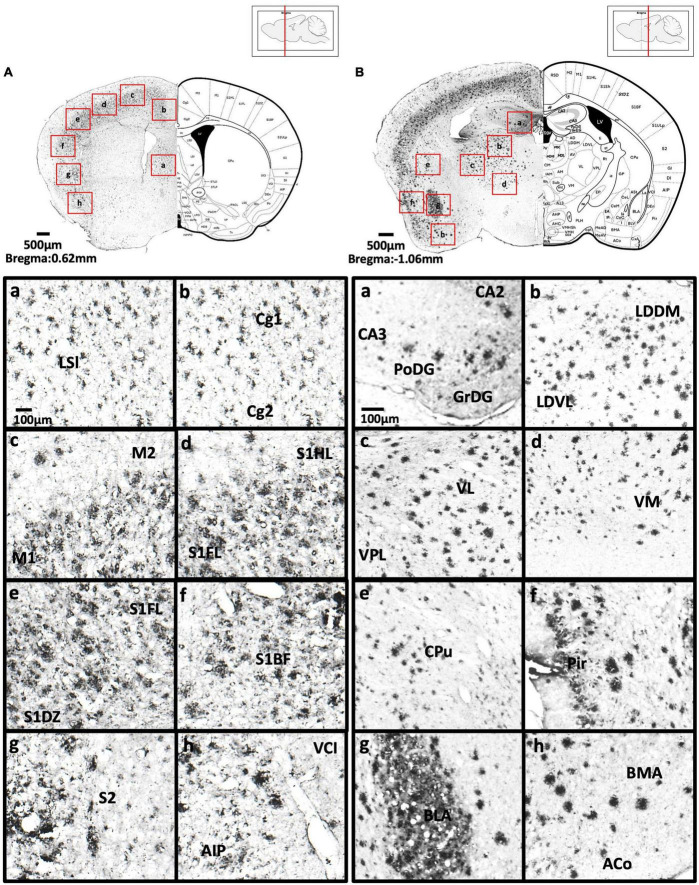
**(A)** Coronal section through the cerebral cortex showing Aβ deposition in the intermediate part of the lateral septal nucleus (a), area 1 and area 2 of the cingulate cortex (b), primary motor cortex and secondary motor cortex (c), hindlimb region and forelimb region of somatosensory cortex (d), forelimb region and dysgranular zone of somatosensory cortex (e), barrel field of the somatosensory cortex (f), secondary somatosensory cortex (g), ventral part of claustrum and posterior agranular insular cortex (h). **(B)** Coronal section through the forebrain showing Aβ deposition in CA2 field, CA3 field and dentate gyrus (a), dorsomedial and ventrolateral areas of the laterodorsal thalamic nucleus (b), ventrolateral thalamic nucleus, ventral posterolateral thalamic nucleus (c), ventromedial thalamic nucleus (d), caudate putamen (e), piriform cortex (f), basolateral amygdaloid nucleus (g), anterior part of the basomedial amygdaloid nucleus, and anterior cortical amygdaloid area (h).

**FIGURE 5:**
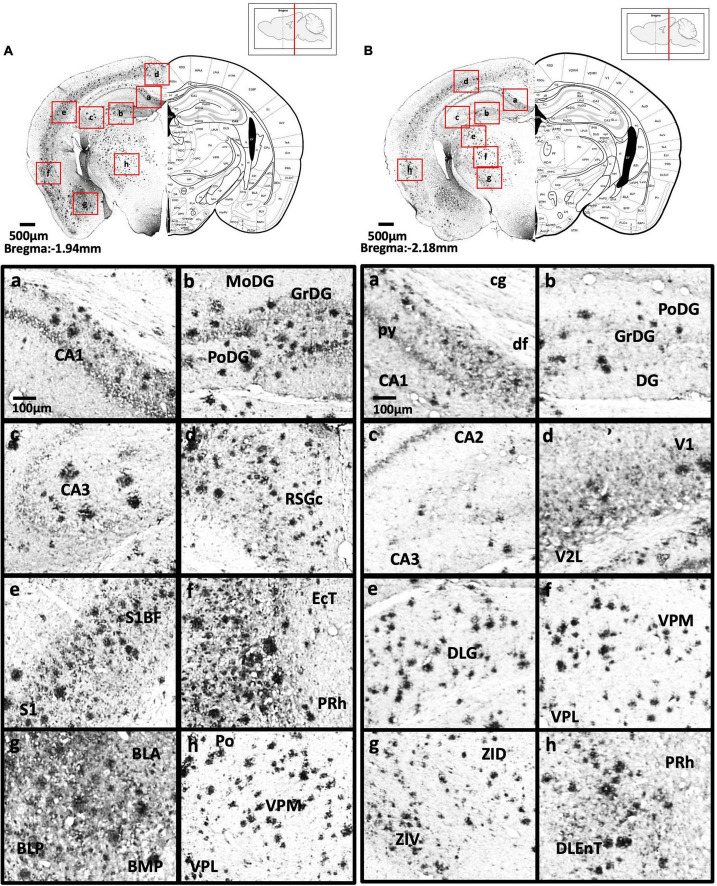
**(A)** Coronal section through the forebrain showing Aβ deposition in CA1 field (a), dentate gyrus (b), CA3 field (c), C region of the retrosplenial granular cortex (d), barrel field of the somatosensory cortex, primary somatosensory cortex (e), ectorhinal cortex, perihinal cortex (f), anterior and posterior basolateral amygdaloid nucleus, posterior basomedial amygdaloid nucleus (g), and posterior, ventral posterolateral and ventral posteromedial thalamus (h). **(B)** Coronal section through the forebrain showing Aβ deposition in the cingulum, pyramidal tract, dorsal fornix and CA1 field (a), dentate gyrus (b), CA2, CA3 (c), primary visual cortex and secondary lateral visual cortex (d), dorsal lateral geniculate nucleus (e), ventral posterolateral and ventral posteromedial thalamus (f), dorsal and ventral part of zona incerta (g), perihinal cortex, and dorsolateral entorhinal cortex (h).

**FIGURE 6:**
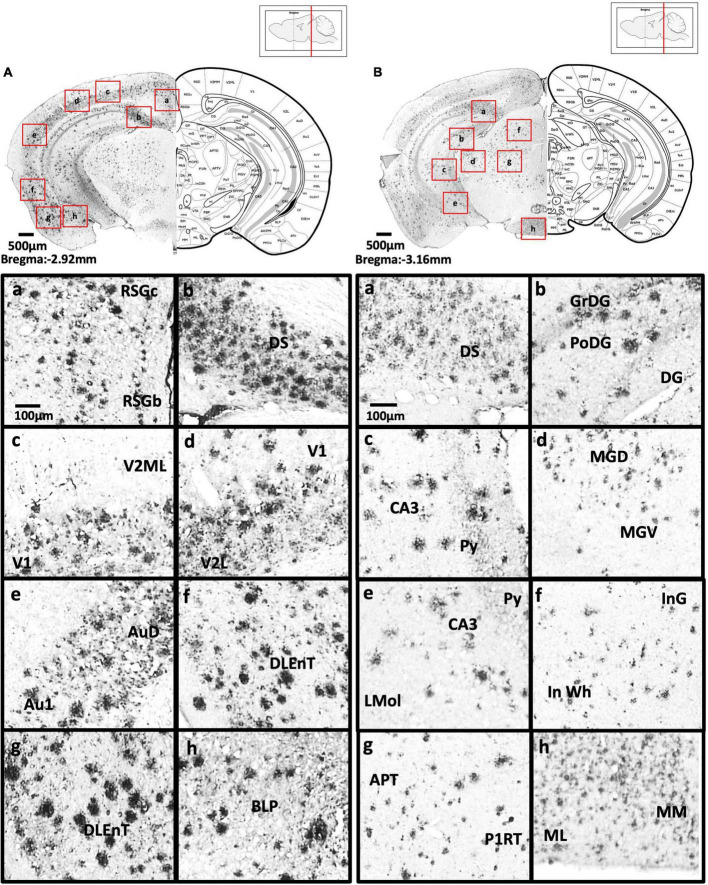
**(A)** Coronal section through the midbrain showing Aβ deposition in the B region and C region of the retrosplenial granular cortex (a), dorsal subiculum (b), primary visual cortex, mediolateral area in the secondary visual cortex (c), primary visual cortex, lateral secondary visual cortex (d), primary auditory cortex, dorsal auditory cortex (e), dorsolateral entorhinal cortex (f), dorsolateral entorhinal cortex (g), and posterior basolateral amygdaloid nucleus (h). **(B)** Coronal section through the midbrain showing Aβ deposition in the dorsal subiculum (a), dentate gyrus (b), CA3 field and pyramidal tract (c), dorsal and ventral medial geniculate nucleus (d), pyramidal tract, CA3 field, and lacunosum molecular layer of the hippocampus (e), intermediate gray layer of the superior colliculus, intermediate white layer of the superior colliculus (f), anterior pretectal nucleus, p1 reticular formation (g), and lateral and medial mammillary nucleus (h).

**FIGURE 7:**
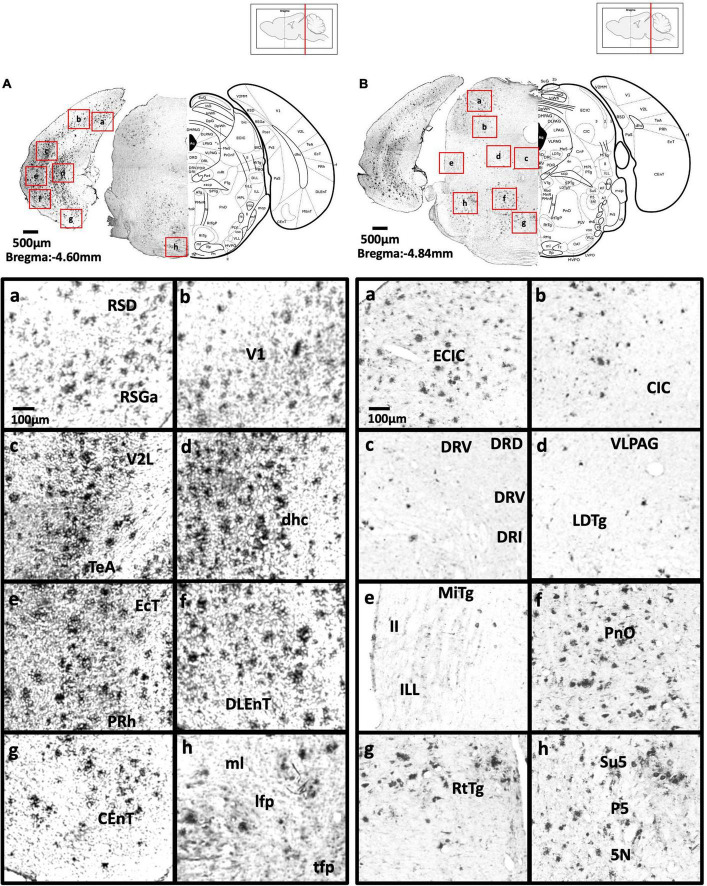
**(A)** Coronal section through the midbrain showing Aβ deposition in the retrosplenial dysgranular cortex, A region of retrosplenial granular cortex (a), primary visual cortex (b), lateral secondary visual cortex (c), dorsal hippocampal commissure (d), ectorhinal cortex, perihinal cortex (e), dorsolateral entorhinal cortex (f), caudomedial entorhinal cortex (g), medial lemniscus, longitudinal fasciculus of nucleus, and transverse fibers of the pons (h). **(B)** Coronal section through the midbrain showing Aβ deposition in the external cortex of the inferior colliculus (a), central nucleus of the inferior colliculus (b), dorsal, ventral, interfascicular, and lateral dorsal raphe (c), ventrolateral periaqueductal gray, laterodorsal tegmental nucleus (d), microcellular tegmental nucleus, lateral lemniscus, intermediate nucleus of the lateral lemniscus (e), oral region of the pontine reticular nucleus (f), reticulotegmental nucleus of the pons (g), supratrigeminal nucleus, peritrigeminal zone, and motor trigeminal nucleus (h).

**FIGURE 8:**
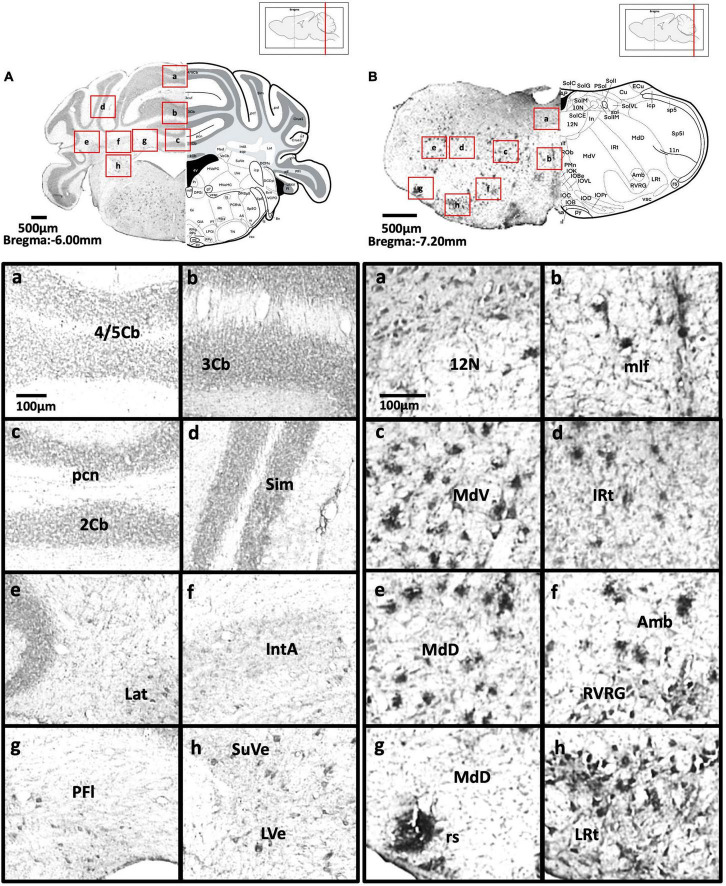
**(A)** Coronal section through the cerebellum and medulla showing Aβ deposition in lobules 4 and 5 of the cerebellar vermis (a), lobule 3 of the cerebellar vermis (b), lobule 2 of the cerebellar vermis and precentral fissure (c), simple lobule (d), lateral cerebellar nucleus (e), anterior interposed cerebellar nucleus (f), paraflocculus (g), superior vestibular nucleus, and lateral vestibular nucleus (h). **(B)** Coronal section through the spinal cord showing Aβ deposition in the hypoglossal nucleus (a), medial longitudinal fasciculus (b), ventral medullary reticular nucleus (c), intermediate reticular nucleus (d), dorsal medullary reticular nucleus (e), ambiguus nucleus and rostral ventral respiratory group (f), rubrospinal tract and dorsal medullary reticular nucleus (g), and lateral reticular nucleus (h).

Figures 2A,B show schematic diagrams of the sagittal view of photomicrographs corresponding to coronal brain sections at different rostro-caudal levels. Figure 2C shows photomicrographs representing intensity levels of Aβ plaque deposition. Representative photomicrographs of coronal brain sections were combined and annotated according to Paxinos and Franklin’s mouse brain atlas (Paxinos and Franklin, 2008; Figures 3–8 and Supplementary Figures 1–3). The abbreviations and terms used in this study can be found in the list of abbreviations in Supplementary Table 1. Qualitative (inter-reliability rate: 0.93) and quantitative assessments of the intensity level of the Aβ plaque load in various ROIs are summarized in Table 1 and Supplementary Table 2, respectively.

**TABLE 1:** Quantitative assessment of the average Aβ plaque deposition in the brain of 5xFAD mice.

Brain regions	Plaque burden (%)±S.D
**Motor cortex**	
Primary Motor Cortex, M1	3.42 ± 1.27
Secondary Motor Cortex, M2	2.75 ± 0.71
**Medial prefrontal cortex**	
Prelimbic Cortex, PrL	1.86 ± 0.69
Infralimbic Cortex, IL	1.86 ± 1.26
**Orbital cortex**	
Lateral, LO	2.59 ± 0.83
Ventral, VO	2.52 ± 0.95
**Agranular insular cortex**	
Ventral/Dorsal, AIV/AID	2.38 ± 1.12
Posterior, AIP	5.94 ± 0.9
**Somatosensory cortex**	
Primary Region, S1	6.61 ± 2.82
Barrel Field, S1BF	5.88 ± 1.30
Forelimb Region, S1FL	6.40 ± 2.21
Hindlimb Region, S1HL	8.76 ± 3.58
Piriform cortex, Pir	6.94 ± 2.00
**Amygdala**	
Basolateral, anterior part, BLA	2.99 ± 0.68
Medial, anterior, MeA	1.22 ± 0.24
Medial, posterior, MeP	2.76 ± 0.98
Medial, Me	1.82 ± 0.40
**Thalamus**	
Posterior, Po	6.82 ± 2.78
Ventral Posterolateral, VPL	7.84 ± 3.20
Ventral Posteromedial, VPM	6.26 ± 2.20
**Dorsal hippocampus**	
CA1 Subfield, CA1	5.56 ± 1.45
CA3 Subfield, CA3	3.69 ± 1.83
Dentate Gyrus, DG	5.16 ± 1.43
**Ventral hippocampus**	
Dorsal Subiculum, DS	18.382 ± 0.93
CA1 Subfield, CA1	8.832 ± 2.76
CA3 Subfield, CA3	9.06 ± 2.86
Dentate Gyrus, DG	6.538 ± 2.92
**Inferior colliculus**	
External Cortex, ECIC	5.19 ± 1.08
**Retrosplenial cortex**	
Dysgranular, RSD	2.3 ± 0.49
**Visual cortex**	
Primary, V1	2.68 ± 0.81
Secondary Lateral, V2L	2.70 ± 0.55
Secondary Mediolateral and Mediomedial Area, V2ML+V2MM	2.29 ± 0.60
**Auditory cortex**	
Primary, Au1	2.18 ± 0.66
Dorsal, AuD	1.99 ± 0.63
Ventral, AuV	1.99 ± 0.53
Temporal association cortex, TeA	1.62 ± 0.37

The plaque burden was measured using the ImageJ threshold method.

#### Olfactory-related areas

We found moderate Aβ deposition in the dorsal, external, lateral, medial, posterior, and ventral regions of the anterior olfactory bulb, olfactory ventricle, dorsal region of the tenia tecta, and the navicular nucleus in 5xFAD mice. We also observed mild Aβ deposition in the glomerular layer and granule cell layer of olfactory bulb, olfactory tubercle, ventral region of the tenia tecta, and dorsal transition zone. However, no Aβ deposition was detected in the olfactory nerve (Figures 3A–C).

#### Anterior cortical area

We found high levels of Aβ deposition in anterior cortical areas in 5xFAD mice, including the perirhinal cortex, primary region (6.61 ± 2.82%), barrel field (5.88 ± 1.3%), forelimb region (6.40 ± 2.21%), dysgranular zone, hindlimb region (8.76 ± 3.58%), jaw region, shoulder region, trunk region, upper lip region, and secondary region of the somatosensory cortex, and piriform cortex (6.94 ± 2.00%). We also observed moderate Aβ deposition in the primary motor cortex (3.42 ± 1.27%), secondary motor cortex (2.75 ± 0.71%), prelimbic cortex (1.86 ± 0.69%), infralimbic cortex (1.86 ± 1.26%), dorsal peduncular cortex of the medial prefrontal cortex, area 1 and area 2 of the cingulate cortex, dorsolateral, lateral (2.59 ± 0.83%) and ventral (2.52 ± 0.95%) regions of the orbital cortex, dorsal and ventral (2.38 ± 1.12%) and posterior regions of the agranular insular cortex (5.94 ± 0.9%), and intermediate part of the endopiriform claustrum. In contrast, only mild Aβ deposition was detected in the medial part of orbital cortex, dorsal and ventral regions of the endopiriform claustrum, and dorsal and ventral claustrum (Figures 3B–4A).

#### Basal ganglia

We observed moderate Aβ deposition in the subthalamic nucleus, nucleus accumbens shell, lateral accumbens shell, and zona incerta; whereas mild Aβ deposition was detected in the pars compacta and pars reticulata of substantia nigra, lateral substantia nigra, nucleus accumbens core, ventral pallidum, caudate putamen, globus pallidus, and internal capsule. However, we did not detect Aβ deposition in the dorsal and medial regions of the substantia nigra pars compacta, or external capsule (Figures 4A–6B).

#### Amygdala and bed nucleus of the stria terminalis

We observed moderate Aβ deposition in the anterior cortical amygdaloid area, posterior basolateral amygdaloid nucleus, anterior basolateral amygdaloid nucleus (2.99 ± 0.68%), medial posterior amygdala (2.76 ± 0.98%), medial anterior amygdala (1.22 ± 0.24%), and amygdalopiriform transition area. We also detected mild Aβ deposition in the anterior amygdaloid area, posterior basomedial amygdaloid nucleus, anterior basolateral amygdaloid nucleus, capsular of central amygdaloid nucleus, extended amygdala, medial amygdala (1.82 ± 0.40%), lateral amygdaloid nucleus, and posteromedial cortical amygdaloid nucleus. However, we found no Aβ deposition in the lateral and medial central amygdaloid or bed nucleus of the stria terminalis (Figures 4B–6B).

#### Thalamus

We found high levels of Aβ burden in the thalamus of 5xFAD mice, particularly in the posterior thalamic nucleus (6.82 ± 2.78%), ventral posterolateral thalamic nucleus (7.84 ± 3.20%), ventral posteromedial thalamic nucleus (6.26 ± 2.20%), and ventrolateral thalamic nucleus. We observed moderate Aβ deposition in the submedius thalamic nucleus, lateral posterior thalamic nucleus, parafascicular thalamic nucleus, and posterior intralaminar thalamic nucleus. We also detected mild Aβ deposition in the rhomboid thalamic nucleus, mediodorsal thalamic nucleus, subparafascicular thalamic nucleus, and ventral anterior thalamic nucleus. However, we did not detect any Aβ deposition in the anterior and posterior paraventricular thalamic nuclei, paratenial thalamic nucleus, or the reuniens thalamic nucleus (Figures 4B–5B).

#### Hypothalamus

We did not detect Aβ deposition in the hypothalamus of 5xFAD mice, including the paraventricular hypothalamic nucleus, striohypothalamic nucleus, posterior, dorsal, ventromedial, dorsomedial, central, and ventrolateral regions, shell region, lateral hypothalamus, lateral tuberal region, ventrolateral part of the hypothalamic area, and medial tuberal nucleus (Figures 4B–5B).

#### Hippocampus

In the dorsal hippocampus of 5xFAD mice, we found high Aβ deposition in dentate gyrus (5.16 ± 1.43%), while moderate Aβ deposition was observed in CA1 (5.56 ± 1.45%), and CA3 (3.69 ± 1.83%) subregions of the dorsal hippocampus. In the ventral hippocampus, high Aβ deposition was shown in dentate gyrus (6.54 ± 2.92%), CA1/2 (8.83 ± 2.76%), CA3 (9.06 ± 2.86%), dorsal, transition area (18.38 ± 0.93%), and ventral subiculum (Figures 4B–6B).

#### Midbrain

In 5xFAD mice, we detected high Aβ burden in the external cortex of the inferior colliculus (5.19 ± 1.08%), whereas moderate Aβ deposition was found in the pontine reticulotegmental nucleus, paranigral nucleus, and intermediate reticular nucleus. We also observed mild Aβ deposition in the dorsal cortex of inferior colliculus, P1, ventrolateral periaqueductal gray, ventral tegmental area, parainterfascicular nucleus, ventral tegmental decussation, lateral superior olive, dorsal nucleus, subnucleus B, and subnucleus C of medial nucleus of the inferior olive, lateral terminal nucleus, supragenual nucleus, and external and rotundus part of the cuneate nucleus. However, we did not detect Aβ deposition in several midbrain regions, including the superficial layer, optic nerve layer, brachium, zona layer, intermediate gray layer, intermediate white layer of superior colliculus, dorsal lateral, dorsal medial, and lateral areas of the periaqueductal gray, caudal, dorsal, interfascicular, lateral, and ventral dorsal raphe, median raphe, nucleus of the trapezoid body, gracile nucleus, gracile fasciculus, median accessory nucleus, and cuneate nucleus of the medulla (Figures 6A–7B).

#### Posterior cortical areas

In 5xFAD mice, we found high Aβ deposition in the dorsal, dorsolateral, and ventral intermediate entorhinal cortex, as well as binocular and monocular areas of the primary visual cortex. We also observed moderate Aβ deposition in the ectorhinal cortex, medial entorhinal cortex, A, B, and C regions of the retrosplenial granular cortex, retrosplenial dysgrandular cortex (2.3 ± 0.49%), primary visual cortex (2.68 ± 0.81%), lateral (2.7 ± 0.55%), mediolateral, and mediomedial (2.29 ± 0.60%) areas of the secondary visual cortex, primary auditory cortex (2.18 ± 0.66%), dorsal (1.99 ± 0.63%), and ventral (1.99 ± 0.53%) areas of the secondary auditory cortex, and the temporal association cortex (1.62 ± 0.37%) (Figures 4B–7B).

#### Cerebellum and vestibular nucleus

We observed mild Aβ deposition in the superior cerebellar peduncle, paraflocculus of the cerebellum, superior, and lateral vestibular nuclei of 5xFAD mice (Figures 8A,B). However, we did not detect Aβ deposition in lobules 2, 3, 4, and 5 of the cerebellum, crus 1 or crus 2 of the ansiform lobule, medial cerebellar nucleus, middle cerebellar peduncle, simple lobule, interposed cerebellar nucleus, lateral cerebellar nucleus, vestibulocerebellar nucleus, medial vestibular nucleus, magnocellular, and parvicellular areas of the medial vestibular nucleus, or spinal vestibular nucleus.

#### Spinal cord and other regions

In 5xFAD mice, we detected moderate Aβ deposition in the gelatinous layer, interpolar, caudal, and oral areas of the spinal trigeminal nuclei, and anterior areas of the anterior commissure. We also observed mild Aβ deposition in the central cervical nucleus of the spinal cord, intrabulbar and posterior nerve of the anterior commissure, magnocellular nucleus of the posterior commissure, nucleus of the posterior commissure, and island of Calleja (Figures 8A,B). We found no Aβ deposition in the nucleus of solitary tract.

#### Correlation

**FIGURE 9:**
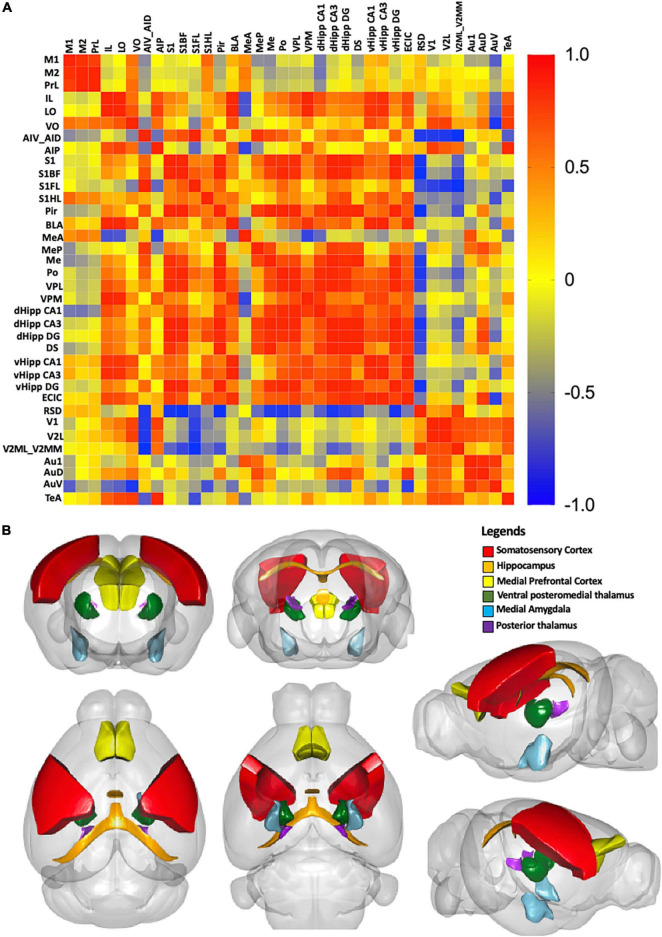
**(A)** Correlation matrix with color scale. A correlation coefficient of 1 represents the maximum positive correlation, whereas, -1 represents the maximum negative correlation. Red indicates 1 and blue indicates -1. **(B)** A 3D model of the strongest correlated regions. Red represents the somatosensory cortex, orange represents the hippocampus, yellow represents the medial prefrontal cortex, darkgreen represents the ventral posteromedial thalamus, and skyblue represents the medial amygdala, purple presents the posterior thalamus.

We examined the possible inter-regional correlation by determining the Aβ plaque burden in the ROIs and their inter-regional correlations in 36 regions. The correlations are presented in the correlation matrix in Figure 9A. Red spots represent strong positive correlation, yellow spots represent no correlation, and blue spots represent strong negative correlation. Distinct red clusters were observed in the medial prefrontal cortex, somatosensory cortex, medial amygdala, thalamus, and hippocampus, which suggest strong relationships among these regions. The 3D model in Figure 9B shows the brain regions with correlation coefficients higher than + 0.9.

## Discussion

In this study, we showed 9-month-old adult 5xFAD mice had significantly impaired spatial learning and memory functions. These results are in line with the memory deficits found in human AD patients (Eustache et al., 2006). As the cognitive impairments seen in the Y-maze and MWM are related to hippocampal-dependent memory formation (Stackman et al., 2003; Kraeuter et al., 2019; Yu et al., 2022), we next investigated the anatomical localization of Aβ deposition in the hippocampus. We found Aβ deposition was strongly associated with the hippocampus and hippocampal-projected regions, including the medial prefrontal cortex, somatosensory cortex, thalamus, and medial amygdala. Our findings are in line with the results from PET studies and human post-mortem brain studies, which observed Aβ deposition in the hippocampus (Martinez-Pinilla et al., 2016; Shokri-Kojori et al., 2018; D’Haese et al., 2020). In AD, Aβ deposition in the hippocampus results in hippocampal atrophy associated with loss of neurons, eventually leading to the accumulation of astrocytes and other glial cells (Martinez-Pinilla et al., 2016; Palmqvist et al., 2017; Uddin and Lim, 2022). Specifically, we found Aβ plaques were widely distributed throughout the dorsal and ventral hippocampus of 5xFAD mice. However, a study on 12-month-old 3xTg-AD mice found Aβ plaque accumulated only in the subiculum of hippocampus (Javonillo et al., 2021). Interestingly, another study on 4-month-old APP/PS1 mice found Aβ plaque accumulation in the hippocampus was associated with cognitive impairment and synaptic marker loss (Sanchez-Varo et al., 2021). These findings and our results not only demonstrate the differential accumulation of Aβ plaques in different AD mouse models, but also the effects of Aβ deposition on cognitive function. We constructed an inter-regional correlation matrix of Aβ deposition, which revealed strong relationships among the hippocampus, medial prefrontal cortex, somatosensory cortex, medial amygdala, and thalamus. The hippocampus together with the regions projecting from it are involved in the control of memory retrieval and consolidation (Hagena et al., 2016). Therefore, the accumulation of Aβ plaques in the hippocampus might also disrupt the functions of these projected areas, contributing to the cognitive impairments.

The deposition of Aβ in the cerebral cortex in AD patients has been confirmed by many studies using polarization sensitive optical coherence microscopy (PS-OCM), positron emission tomography (PET), and the magnetic resonance imaging (MRI) (Klunk et al., 2004; Davatzikos et al., 2008; Johnson et al., 2012; Baumann et al., 2017). Moreover, a PET scan study in humans revealed that the first region to experience Aβ deposition was the cerebral cortex, particularly the precuneus, medial orbitofrontal, and posterior cingulate cortices (Palmqvist et al., 2017). In 5xFAD mice, Aβ deposition was found to start in the subiculum of the hippocampus and layer V of the cerebral cortex by 2 months of age (Oakley et al., 2006). In our study, the immunostaining not only showed pronounced Aβ deposition throughout the cerebral cortex of 5xFAD mice, but also revealed two distinct Aβ deposition patterns in the cerebral cortices. We detected Aβ deposition in layers 4, 5, and 6a (somatosensory cortex, visual cortex, and auditory cortex) and in layers 1, 2, and 3 (agranular insular cortex and piriform cortex) of 5xFAD mice. Similar findings were observed in previous studies that showed neuronal loss in cortical layers 4 and 5 in APP/PS1 and 5xFAD mice, respectively (Beker et al., 2012; Jawhar et al., 2012; Eimer and Vassar, 2013). In this study, we are first to demonstrate an Aβ deposition pattern in cortical layers 1, 2, and 3 of 5xFAD mice.

In the correlation matrix, the medial prefrontal cortex and somatosensory cortex showed strong correlations with the hippocampus. The cerebral cortex is highly involved in hippocampal-dependent memory formation, given that it receives numerous projections from the hippocampus. The projection from the hippocampus to medial prefrontal cortex regulates social, hippocampal-dependent and fear memory (Lim et al., 2015a; Liu et al., 2015; Tan et al., 2020a,^2021^), whereas the projection from hippocampus to the somatosensory cortex regulates somatosensory responses and the consolidation of human motor memory (Bellistri et al., 2013; Kumar et al., 2019). Moreover, projections from the hippocampus to other cortical areas, such as the retrosplenial cortex, are involved in the retention of recognition memory (Balcerek et al., 2021). The hippocampus also sends navigational signals to visual cortex to induce a visual response, and to the auditory cortex to modulate auditory information and auditory fear conditioning (Xiao et al., 2018; Diamanti et al., 2021). Therefore, Aβ deposition in the hippocampus and cerebral cortex might further increase cognitive impairment.

We observed moderate to mild Aβ deposition in the amygdala, which showed a strong positive correlation with the hippocampus in the correlation matrix. The medial amygdala is involved in innate emotional behavior *via* regulating olfactory information to hypothalamus (Lim et al., 2008; Lim et al., 2009; Keshavarzi et al., 2014). Therefore, Aβ deposition in medial amygdala might lead to altered emotional behavior. On the other hand, the projection from the hippocampus to the amygdala also regulates the formation of long-term memories, particularly significant emotional events (Richter-Levin and Akirav, 2000). Remarkably, human AD patients were shown to have impaired amygdala-dependent memory, in which they failed to exhibit conditioned fear responses to stimuli (Hamann et al., 2002). Consistently, Aβ deposition induced impairment of amygdala-dependent memory in AD animal models, including APPswe/PS1dE9, APP(Ind)/APP(Sw, Ind), and 3xTg-AD transgenic mice (Espana et al., 2010; Lin et al., 2015).

We detected mild Aβ deposition in the pars compacta and pars reticulata of substantia nigra and ventral tegmental area (VTA). These regions also receive projections layer V of the cortex layer V of the cortex from the hippocampus and are involved in hippocampal memory systems (Martig et al., 2009; Kafkas and Montaldi, 2015). The VTA and substantia nigra are two major dopaminergic areas in the brain. The deposition of Aβ in these regions can disrupt dopaminergic-regulated reward memory processing in 3xTg-AD and Tg2576 mouse models (Nobili et al., 2017; Gloria et al., 2021). Moreover, tyrosine hydroxylase cells and dopamine neurons in the VTA and substantia nigra were significantly suppressed in 5xFAD mice (Vorobyov et al., 2019). In human study, there were remarkable reduction of catecholamine and their metabolites (Liu et al., 2011), as well as alteration of dopamine and its receptors in AD patients (Pan et al., 2019).

We are first to show mild Aβ deposition in both the VTA and substantia nigra of 5xFAD mice, which suggests mild Aβ deposition may be sufficient to suppress the dopaminergic system and disrupt memory encoding and memory consolidation processes in 5xFAD mice, although the dopaminergic system could also be affected by other AD pathologies. Our study also demonstrated for the first time mild to moderate Aβ deposition in the nucleus accumbens of 5xFAD mice. The nucleus accumbens is a key regulator of reward and satisfaction, and also receives projections from the hippocampus to mediate decision-making processes (Abela et al., 2015; Salgado and Kaplitt, 2015) and impulsivity (Sesia et al., 2008; Sesia et al., 2010). It was found that Aβ accumulation in the nucleus accumbens suppressed cholinergic, dopaminergic, and norepinephrine systems in Wistar rats (Preda et al., 2008; Morgese et al., 2015).

We found Aβ deposition in various regions of the thalamus of 5xFAD mice. Deposition of Aβ in thalamus was also reported in post-mortem human AD brain tissues and in AD models by positron emission tomography (Alderson et al., 2017; Frost et al., 2020). The thalamus is connected to the hippocampus to regulate spatial memory, spatial sensory information, and human episodic memory (Burgess et al., 2002; Aggleton et al., 2010; Chan et al., 2017). Therefore, Aβ deposition in the thalamus might contribute to the cognitive impairment in AD (Alderson et al., 2017; Frost et al., 2020).

We did not detect Aβ deposition in the hypothalamus of 5xFAD mice, which is contrary to the report of Aβ deposition in the paraventricular nucleus of the hypothalamus in autopsy brain of AD patients (Ishii and Iadecola, 2015). The hypothalamus contains many nuclear divisions that regulate neuroendocrine systems and serve as the primary coordinator of memory updating (Clarke, 2015; Burdakov and Peleg-Raibstein, 2020). It was reported that AD patients have increased basal cortisol levels, overall insensitivity to glucocorticoid feedback, lowered thyroid hormones, and gradual decline of estrogen and testosterone (Ishii and Iadecola, 2015). The altered glucose metabolism might be one of the possible reasons that caused the absent of Aβ in hypothalamus. A study demonstrated hypothalamus as a primary brain region with metabolic abnormalities in APP/PS1 transgenic mouse (Zheng et al., 2018). A reduced cerebral glucose uptake pattern was observed in 5-month-old 5xFAD mice, and a more significant reduction was observed in the 13-month-old mice. Tharp et al. (2016) showed the correlation between glucose level and Aβ secretion. Therefore, glucose metabolism in the hypothalamus of 5xFAD mice warrants further investigation to justify the lack of Aβ in this region.

Although we observed Aβ deposition throughout the olfactory areas except for the olfactory nerve, the effect of the Aβ deposition on olfactory memory was ambiguous. Two studies suggested that 5xFAD mice retain intact olfactory memory from 3 to 15 months of age without olfactory impairment (Girard et al., 2014; O’Leary et al., 2020), although another study showed that 3-month-old 5xFAD mice did not have full olfactory function (Son et al., 2021). Other studies observed olfactory dysfunction in human AD patients, 3xTg-AD mice, and APPxPS1 mice (Saiz-Sanchez et al., 2013; Franks et al., 2015; Mitrano et al., 2021). Further studies with more stringent behavioral testing are needed to reveal whether Aβ deposition has negative effects on olfactory memory in 5xFAD mice.

We also did not detect Aβ deposition in the periaqueductal gray of 5xFAD mice, which contradicts the findings in a post-mortem AD brain study that observed senile plaques in the periaqueductal gray in 81% of samples (Parvizi et al., 2000). The periaqueductal gray is a major component of brainstem that has pivotal roles in autonomic function, behavior, memory formation, and emotional response to aversive events (Lim et al., 2008; Lim et al., 2010; Temel et al., 2012; Tan et al., 2020b). The absence of Aβ deposition in the periaqueductal gray in 5xFAD mice might represent an inability to recapitulate the Aβ pathological characteristics of human AD.

In summary, the present findings provide comprehensive insights on the rostral-caudal distribution profile of Aβ depositions and their inter-regional correlation in adult 5xFAD mouse brain. Although our study focused on Aβ deposition, other hallmarks of AD (e.g., neurofibrillary tangles, presenilin, and apolipoprotein E) are equally important and will need to be investigated to further enhance translation from animal models to clinical applications.

## Data availability statement

The raw data supporting the conclusions of this article will be made available by the authors, without undue reservation.

## Ethics statement

The animal study was reviewed and approved by Committee on the Use of Live Animals in Teaching and Research, The University of Hong Kong (No. 4807-18).

## Author contributions

TS, YS, MLF, and LWL: conceptualization, funding acquisition, project administration, resources, and supervision. KCT, JR, SCC, and LS: data curation and formal analysis. KCT, JR, SCC, KHW, LS, CHP, YW, TS, LA, RCCC, YS, MLF, and LWL: investigation. KCT, JR, SCC, YS, MLF, and LWL: methodology. KCT, JR, SCC, LA, and KHW: visualization and validation. KCT and LWL: writing–original draft. JR, SCC, KHW, LS, CHP, YW, TS, LA, RCCC, YS, and MLF: writing–review and editing. All authors have read and agreed to the published version of the manuscript.

## Conflict of interest

The authors declare that the research was conducted in the absence of any commercial or financial relationships that could be construed as a potential conflict of interest.

## Publisher’s note

All claims expressed in this article are solely those of the authors and do not necessarily represent those of their affiliated organizations, or those of the publisher, the editors and the reviewers. Any product that may be evaluated in this article, or claim that may be made by its manufacturer, is not guaranteed or endorsed by the publisher.
