# Supplementary material for: Distribution and inter-regional relationship of amyloid-beta plaque deposition in a 5xFAD mouse model of Alzheimer’s disease
Source: Front Aging Neurosci. 2022 Jul 28;14:964336. doi: 10.3389/fnagi.2022.964336 (PMC9371463; doi:10.3389/fnagi.2022.964336)

**Supplementary Table 1.** Abbreviation list of brain structures in alphabetical order.

| 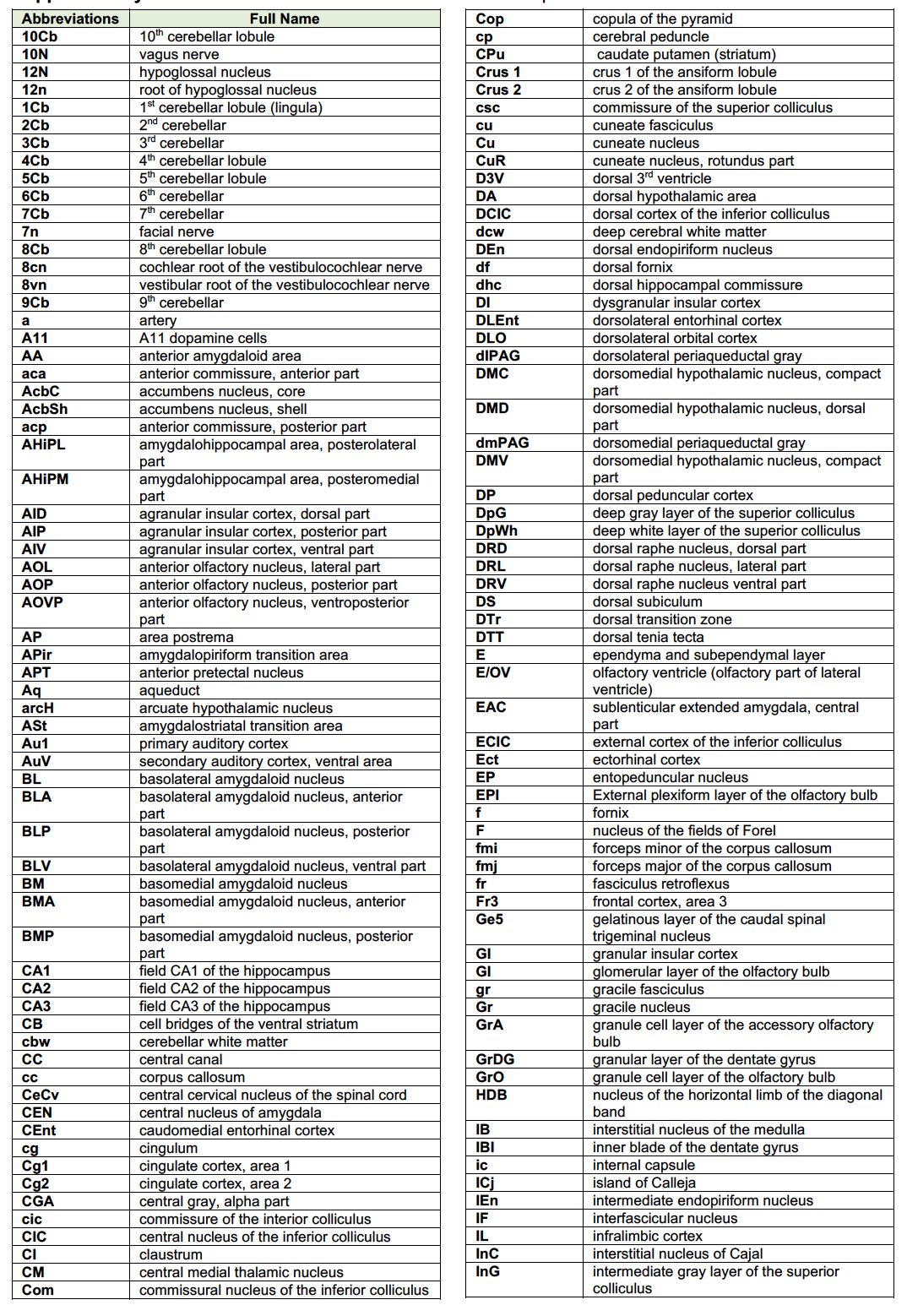 | 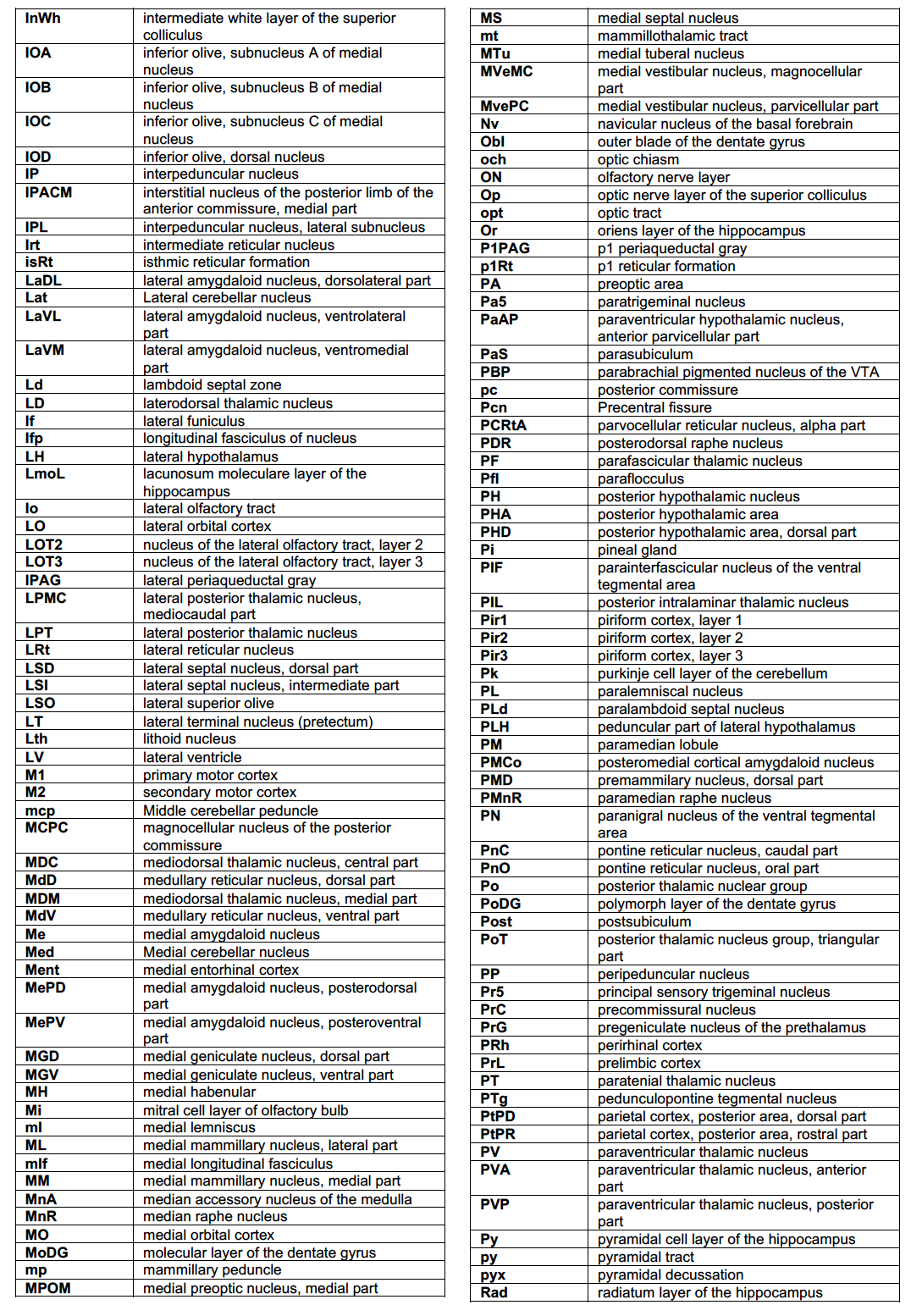 | 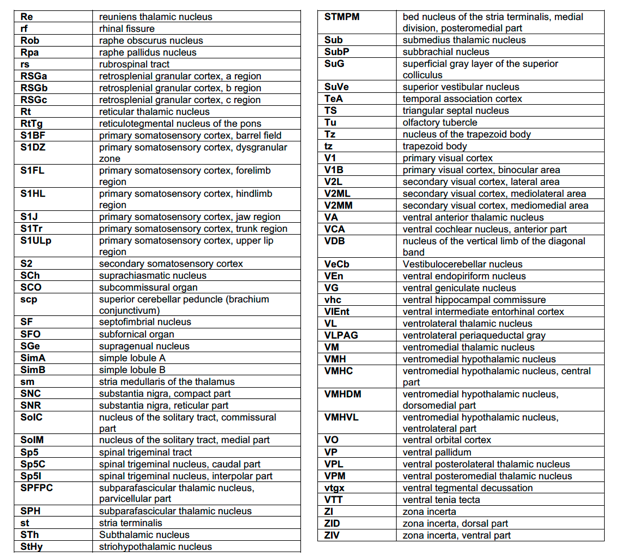 |
| --- | --- | --- |

**Supplementary Table 2.** Qualitative assessment of the average Aβ plaque deposition in the brain of 5xFAD mice. The intensity average was rated by two researchers according to the intensity scale (0 = no deposition; 1 = mild; 2 = moderate; 3 = high).


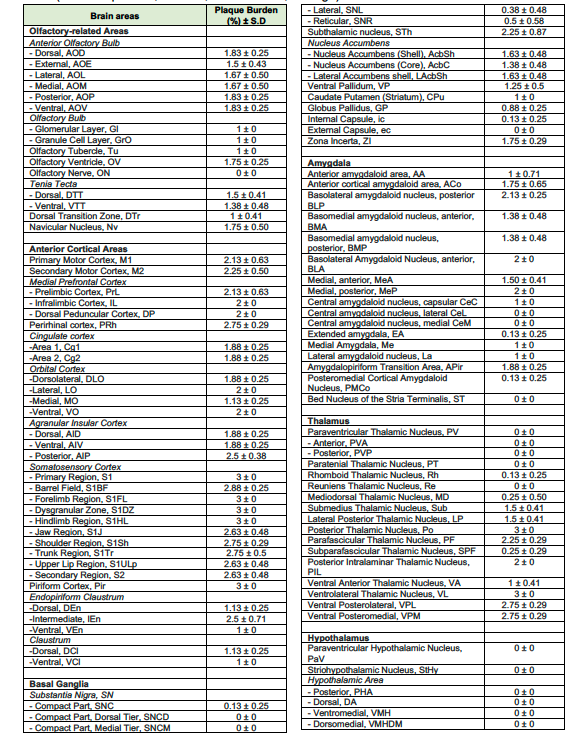

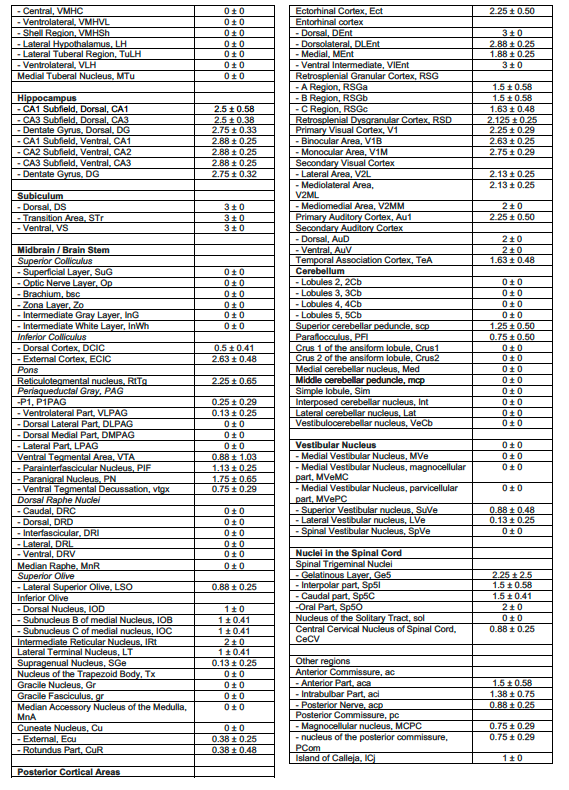

Supplement: Supplementary file 1 [file Table_1.DOCX]
