# Supplementary figures and images for: Distribution and inter-regional relationship of amyloid-beta plaque deposition in a 5xFAD mouse model of Alzheimer’s disease
Source: Front Aging Neurosci. 2022 Jul 28;14:964336. doi: 10.3389/fnagi.2022.964336 (PMC9371463; doi:10.3389/fnagi.2022.964336)

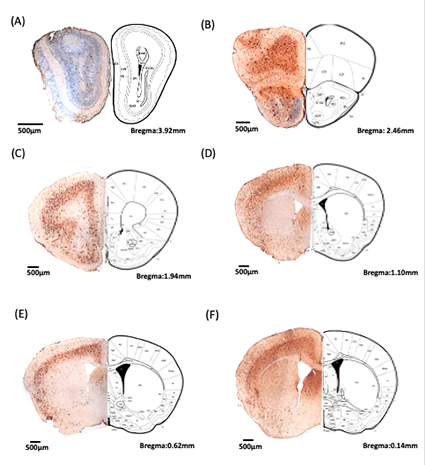

Supplement: Supplementary file 2 [file Image_1.PNG]

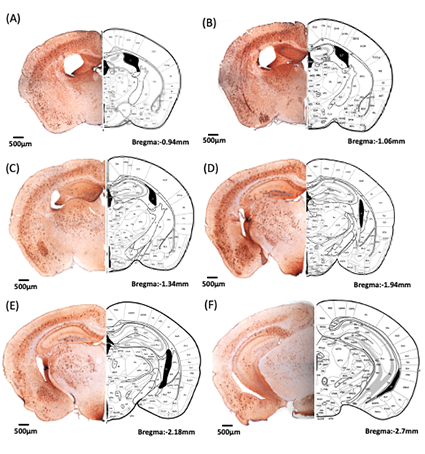

Supplement: Supplementary file 3 [file Image_2.PNG]

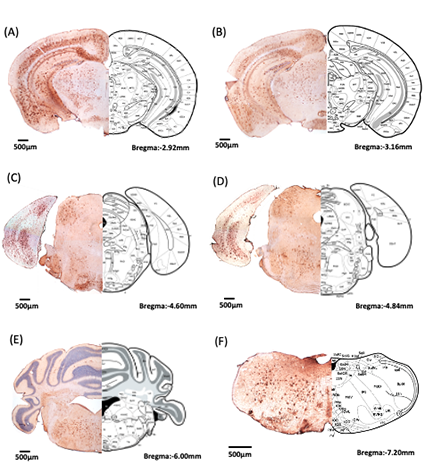

Supplement: Supplementary file 4 [file Image_3.PNG]
